# Prolonged Feedback Duration Does Not Affect Implicit Recalibration in a Visuomotor Rotation Task

**DOI:** 10.1523/ENEURO.0447-21.2022

**Published:** 2022-04-19

**Authors:** Jana Maresch, Opher Donchin

**Affiliations:** 1 Department of Brain and Cognitive Sciences, Ben Gurion University of the Negev, Be'er Sheva 8410501, Israel; 2 Department of Biomedical Engineering and Zlotowski Center for Neuroscience, Ben‐Gurion University of the Negev, Be'er Sheva 8410501, Israel

**Keywords:** implicit recalibration, motor adaptation, visuomotor rotation

## Abstract

Visuomotor rotations are frequently used to study cognitive processes underlying motor adaptation. Explicit aiming strategies and implicit recalibration are two of these processes. A large body of literature indicates that both processes are in fact dissociable and mainly independent components that can be measured using different manipulations in visuomotor rotation tasks. Visual feedback is a crucial element in these tasks, and it therefore plays an important role when assessing explicit re-aiming and implicit recalibration. For instance, researchers have found timing of visual feedback to affect the contribution of implicit recalibration to learning: if feedback is shown only at the end of the movement (instead of continuously), implicit recalibration decreases. Similarly, participants show lower levels of implicit recalibration if visual feedback is presented with a delay (instead of immediately). We thus hypothesized that the duration of feedback availability might also play a role. The goal of this study was thus to investigate the effect of longer versus shorter feedback durations on implicit recalibration in human participants. To this end, we compared three feedback durations in a between-subject design: 200, 600, and 1200 ms. Using a large sample size, we found differences between groups to be quite small, to the point where most differences indicated statistical equivalence between group means. We therefore hypothesize that feedback duration, when only endpoint feedback is presented, has a negligible effect on implicit recalibration. We propose that future research investigate the effect of feedback duration on other parameters of adaptation, so as proprioceptive recalibration and explicit re-aiming.

## Significance Statement

Knowledge about explicit and implicit processes in motor adaptation forms a crucial aspect in our understanding of motor learning in general. We know that the smallest changes in visual feedback might lead to more or less implicit recalibration, which in turn can lead to changes in performance. One aspect, that has not yet been tested, is whether duration of visual feedback affects implicit recalibration. We find that changes in implicit recalibration are very small, if they exist at all. This is an important finding which extends our knowledge about the factors that do or do not influence implicit recalibration.

## Introduction

The ability to adapt our behavior to environmental perturbations is an important function of our nervous system. When faced with a systematic perturbation, such as encountering wind when playing badminton on an outdoor court, our brain will gradually adjust our motor output to minimize the resulting error. This gradual, implicit recalibration originates from a mismatch between what our sensory organs tell us about where our body (or the shuttlecock in our example) moved and what our brain predicted they would tell us, a phenomenon called sensory prediction error (Miall and Wolpert, 1996; Tseng, Diedrichsen et al., 2007; Shadmehr, Smith et al., 2010). However, when seeing the shuttlecock deviate to the left unexpectedly, we will also strategically aim farther to the right on the next hit. Our actual motor output will therefore be some combination of implicit recalibration and explicit re-aiming (Taylor et al., 2014; Heuer and Hegele, 2015; McDougle et al., 2015). In a lab setting, these processes are often studied using visuomotor rotation tasks, where visual representation of hand position is perturbed so that subjects must learn a new mapping of motor commands. Since the only visual feedback participants receive is via this visual representation, typically a cursor on the screen, this task provides the perfect ground to investigate how visual feedback influences implicit recalibration and explicit re-aiming. An important factor seems to be the timing of visual feedback regarding the outcome of the reaching movement. Several studies, for example, compared conditions in which visual feedback is available throughout the entire movement to conditions in which feedback is presented only around movement termination (Shabbott and Sainburg, 2010; Schween et al., 2014; Taylor et al., 2014; Bond and Taylor, 2015). These studies consistently found a reduction in implicit recalibration when visual feedback was present at the end of the movement only. Schween and Hegele (2017) found that the timing of the feedback also plays an important role in the modulation of the implicit process. In their study, the authors assessed the effect of delayed terminal visual feedback on implicit recalibration. They found that longer, as opposed to shorter or no, feedback delays result in less implicit recalibration (Schween and Hegele, 2017). Thus, adaptation becomes more implicit when visual feedback is limited – this is true with continuous feedback and endpoint feedback and also with longer and shorter feedback delays. Most studies using endpoint feedback use the same, relatively long, feedback durations (1000–1600 ms; Schween et al., 2014; Taylor et al., 2014; Bond and Taylor, 2015; Schween and Hegele, 2017; de Brouwer et al., 2018). These are long durations, even longer than in online feedback conditions, these times are generally the same as the associated movement times and vary between 400 and 600 ms. This suggests that further limiting availability of visual feedback by shortening the time feedback is presented at the end of the movement would reduce implicit recalibration even more. In order to test this idea, we exposed participants to a standard, abrupt visuomotor rotation and gave different feedback durations, 200, 600, and 1200 ms, to different groups of subjects. We chose 200 ms as our shortest feedback duration because we wanted to ensure that participants were able to consciously process the sensory stimulus (Esteves and Öhman, 1993). The two longer durations were chosen to see whether there is a gradual effect of feedback duration on implicit recalibration.

## Materials and Methods

### Participants

A total of 78 right-handed subjects participated in the study and were randomly assigned to one of three feedback duration groups: the short-duration group (*n* = 27; mean age: 25 [23–28]; seven females), the medium-duration group (*n* = 25; mean age: 25 [22–28]; 12 females) and the long-duration group (*n* = 25; mean age: 24 [22–27]; 14 females). All subjects signed an informed consent form, which included basic information about their relevant medical status. Subjects were not included for participation if they had previously participated in visuomotor rotation research, if they had any neurologic disorders or if they suffered from vertigo. Subjects were contacted and recruited through the department of Biomedical Engineering and the department for Brain and Cognitive Sciences and received monetary compensation for their participation. The experimental protocol was approved by the Human Subject Research Committee of the Ben Gurion University of the Negev and followed the ethical guidelines of the university.

### Apparatus

Participants were seated on a height-adjustable chair, facing a 24-inch, 60-Hz LCD screen positioned approximately at head height 50 cm in front of them. We chose a frontoparallel screen set-up to stay close to the set-up used by Schween and Hegele (2017), since this is one of the key studies driving our hypothesis. A square occluder 20 cm above the table prevented vision of the hand. On the screen, subjects saw a cursor and targets to move to (detailed in the next section, Design and procedure). Using a digital stylus, participants made center-out, horizontal reaching movements on a digitizing Wacom Intuos Pro tablet with an active area of 311 × 216 mm and a polling rate of 2154 Hz. Data collection and stimulus presentation were controlled by custom scripts in MATLAB (R2020b).

### Design and procedure

**Figure 1.**
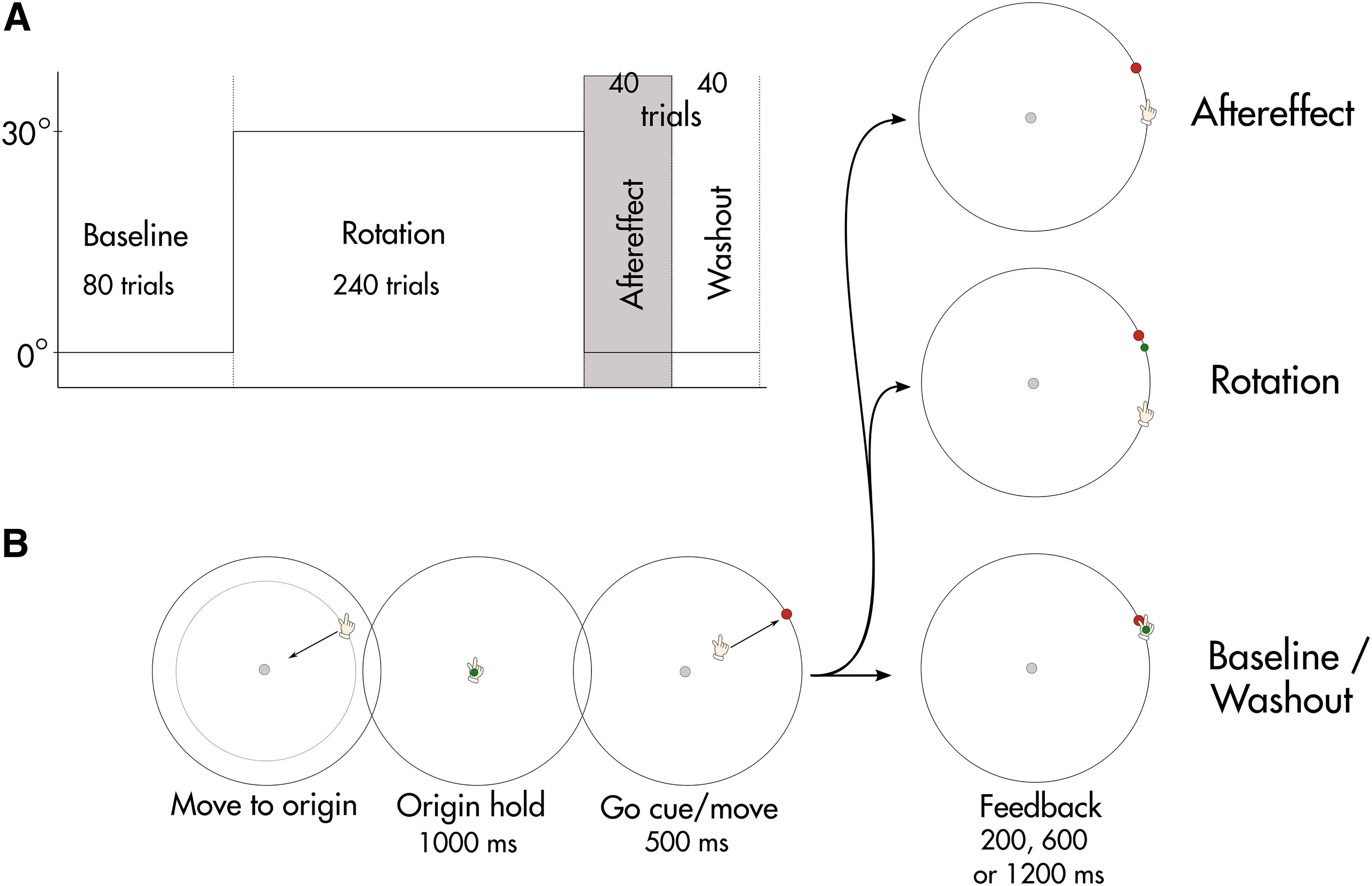
Experimental setup and design. ***A***, The experiment is made up of four different phases: baseline, rotation, aftereffect, and washout. ***B***, Trial structure. Each trial started with the participants moving their hand into the starting position (filled gray dot), where they stayed for 1000 ms (origin hold) until the target appeared (filled red dot). They had 500 ms to execute the movement and received visual feedback for 200, 600, or 1200 ms depending on their assigned group (shown as filled green dot). Visual feedback could be either rotated (Rotation) or not present (Aftereffect).

The experiment was made up of four different phases, as shown in Figure 1*A*: a baseline phase with veridical cursor feedback (80 trials), a rotation phase with rotated cursor feedback (240 trials), an aftereffect phase without any visual cursor feedback (40 trials), and a washout phase with veridical cursor feedback (40 trials). During the rotation phase, a rotation of 30° was applied to the cursor, such that when the cursor reappeared at the end of the trial, it was displaced by 30° in the clockwise direction relative to the hand. Note that participants were not informed about the rotation. Instead, they were told that the task will become more difficult at some point and instructed to continue to try and slice the target with their cursor. Before the aftereffect phase, participants were informed that they would not receive any visual feedback in the following block of trials and they were instructed to move their hand straight to the target and stop using any strategy they might have been using previously. In the final washout phase, visual cursor feedback was restored in the same format as in the initial baseline block.

### Trial structure

The trial structure is shown in Figure 1*B*. Participants reached to targets in different directions from the origin in the center of the screen, which was marked by a white disk (5-mm radius). Each trial started with participants moving toward the origin. A white ring indicated the distance from the current hand position to the origin and helped to guide the participants to their start position without providing information about their exact hand location. The ring became progressively smaller the closer participants got to the origin. When the hand was 0.56 cm from the origin, a small circular cursor (green, 4-mm radius) appeared in the exact hand location, allowing participants to move their cursor into the center of the origin. The respective target location appeared after participants stayed in the origin for 1000 ms. It was shown as a red filled circle (5-mm radius) 8 cm from the origin, which corresponded to a movement of 5.5 cm on the tablet (in the following text we will refer to distances on the screen). Target locations varied across trials and could appear on a circle of eight possible, equally spaced locations in cardinal directions (22.5° between targets, with 0° corresponding to horizontal rightward movement and a negative sign indicating counterclockwise rotation). Targets were presented in a pseudorandom order such that all targets were experienced once before being repeated in the next cycle. The visual cursor reappeared at the end of each trial when the hand crossed a distance of 7.6 cm (95% of the target distance) from the origin. When the cursor reappeared, it was shown as stationary feedback for a duration of 200, 600, or 1200 ms, depending on the group. Participants were instructed to make fast and accurate shooting movements to the target, “slicing” through the target. Once they stopped their movement, they were required to remain in the same position until the feedback disappeared. They had 500 ms to reach the distance of 7.6 cm from their start position and were rewarded by a pleasant “ding” sound if they managed to do so. Failure to comply with these timing criteria resulted in an unpleasant “buzz.”

### Movement analysis

For offline analysis, we defined movement onset as the moment when the hand crossed 10% of the peak velocity of the respective trial. Movement end was defined as the first moment after peak velocity when the hand movement was slower than 10% of the peak velocity of the respective trial. Our primary outcome measure was the movement direction at target distance during early aftereffect (used as our estimate of implicit recalibration). We calculated the movement direction for all trials as the angular difference between the vector connecting the start location with the respective target and the vector connecting the start location with the hand position at target distance. All trajectories were rotated to a common axis with the target location at 0°. Positive angles indicate a counterclockwise deviation of the hand from the target, meaning a clockwise rotation of the cursor. Reaction time for all groups was defined as the time between target appearance and movement onset as used in previous reports (Taylor et al., 2014; Bond and Taylor, 2015; McDougle et al., 2015). Trials in which distance from the origin was not strictly increasing after the cursor passed a distance of 0.4 cm from the origin and trials in which movements did not reach a distance of 7.6 cm from the origin were excluded from the analysis. As a consequence, ∼1% trials were excluded from further analysis.

### Statistical analysis

**Figure 2.**
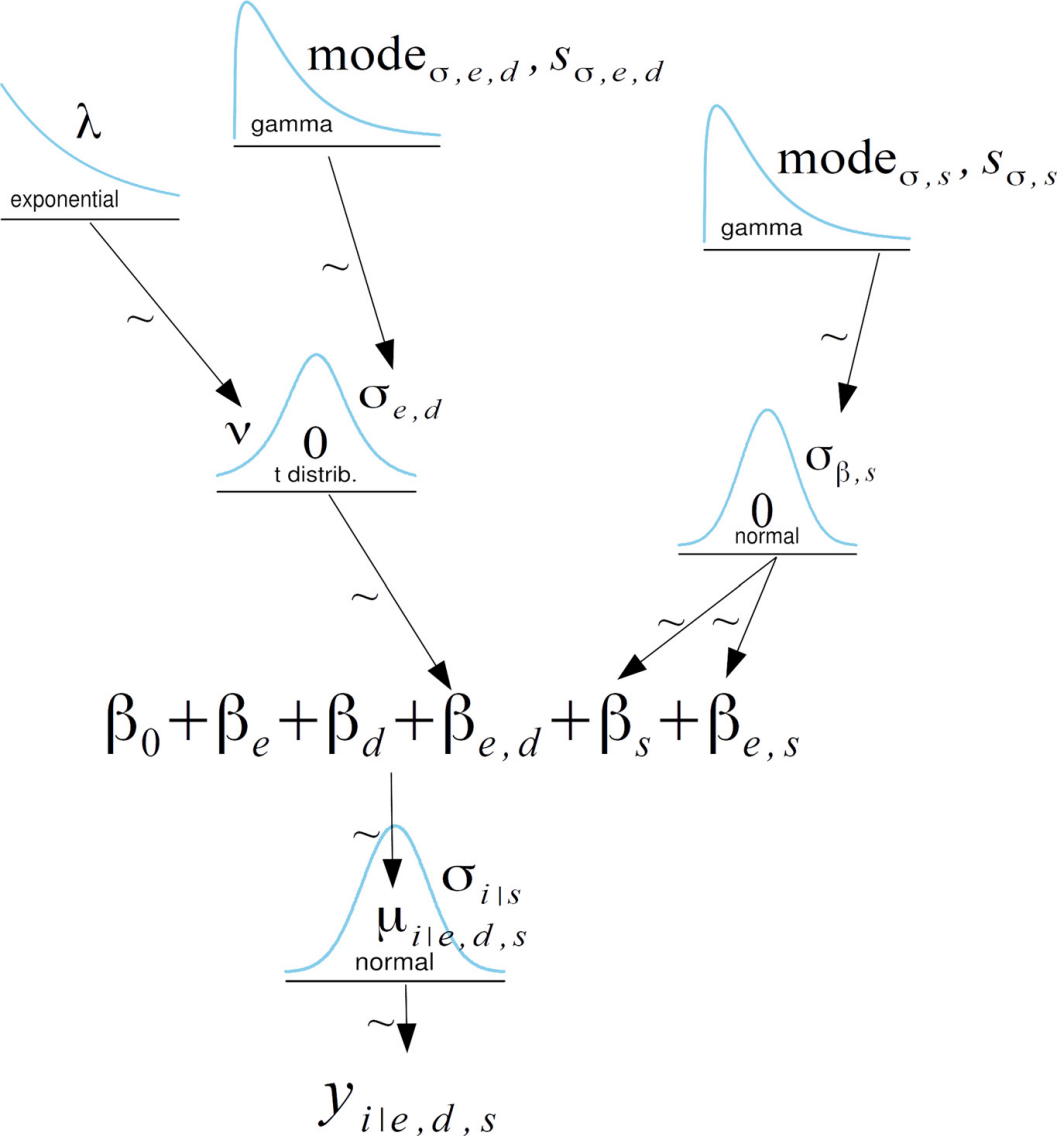
Simplified diagram of the hierarchical Bayesian model showing how the key coefficients for our statistical comparison were determined.

We used a Bayesian statistical approach very similar to the approach used by Maresch et al. (2020) to fit a linear ANOVA model to our data. The Bayesian approach combines prior information about population parameters with evidence contained in the data to find the posterior probability distributions for the parameters given the data (Kruschke, 2014). In contrast to frequentist approaches, Bayesian probabilities are thus statements about probabilities of the parameters rather than the sampling distribution of the estimators. Full details of the model are available in the online repository (https://osf.io/f6smc/), and here we describe the key points only. The dependent variable for our linear model was the movement direction of each subject on each trial. We separated the different phases of the experiment into a series of epochs containing different numbers of trials (Table 1). The model’s independent variables included the epoch type, the subject’s group, and the subject id. The model also included the group by epoch type interaction and the subject id (a unique number identifying each participant). A simplified diagram of the model we used is presented in Figure 2. The figure emphasizes two key aspects of the model. First, the coefficients representing the episode by duration interaction (the key coefficients for all of the statistical comparisons in this study) were drawn hierarchically from a *t* distribution. This allowed for specific episode/duration combinations to be outliers relative to the others, key for identifying effects in the data. The second aspect of the model that bears noting is that the “random effect” of subjects was also estimated hierarchically. This means that we estimated subject-specific intercept and interaction values in a way that regresses toward the mean. Prior distributions were chosen to be essentially uniform across a range that includes all reasonable values of the model’s parameters. Changing these parameters by a factor of 2 did not change the posterior distribution in any meaningful way. We assumed that the standard deviation of the movement direction differed among subjects and was sampled as a parameter of the hierarchical model with a γ prior.

**Table 1:** Overview of the different epochs

Epoch types	Number of epochs	Number of trials per epoch	Total number of trials
Early baseline	1	40	40
Late baseline	1	40	40
Adaptation	30	8	240
Early aftereffect	1	20	20
Late aftereffect	1	20	20
Washout	1	40	40

Shown are number of epochs as used in the Bayesian analysis and the number of trials included in each epoch.

The joint posterior distribution of the model’s parameters was sampled using JAGS (4.3.0; http://mcmc-jags.sourceforge.net/) called from MATLAB (2020b, the MathWorks) using matjags (https://github.com/msteyvers/matjags/blob/master/matjags.m). We used four chains, 1000 burn in samples and 10,000 samples per chain. Using standard diagnostics described by Kruschke (2014), we ensured that the chains converge to a unimodal distribution for all parameters and that the results were consistent across chains. We then calculated the posterior distribution of the average movement direction for each group for the different epochs (see Table 1) from the sampled estimates of the regression coefficients. The posterior distributions gave us estimates of credible values given our initial model. Next, we calculated the high-density intervals (HDIs) from the posterior distributions for each group for all epochs and for the differences between groups for our main outcome measure, early aftereffect.

We report our results using the mean and 95% HDI of the mean. The HDI contains 95% of the distribution, in which every point has higher credibility than any point outside this range. Furthermore, for group comparisons we specified a region of practical equivalence (ROPE) of −3° through 3° around a value of 0 difference between the groups. We chose this ROPE based on what we consider a meaningful difference, however, since we report all actual differences in our results, each reader can decide for themselves what they consider a meaningful difference. We report the percentage of the HDI that lies within the ROPE as a measure of the probability that the values are equivalent.

Unless otherwise indicated, brackets represent 95% HDI.

### Sample size estimation

After collecting an initial dataset of 35 subjects, we estimated the number of subjects necessary to reach HDIs for the group means that were <2°. We did this to determine whether it was feasible to use the precision of our estimates as our stopping criterion. This criterion is held to yield the least bias and low error rates compared with other stopping rules (Kruschke, 2014; Kruschke and Liddell, 2018). It has the advantage that final sample size is determined by how well one is able to characterize the effect rather than using arbitrary criteria. Specifically, we chose a criterial-precision stopping rule of an HDI width per group of 2° and an HDI width for group differences of 2.5°. Thus, if the HDI width for group differences was 2.5° or less, and group differences continued to be within the ROPE, chances were very high that there was no difference between groups.

**Figure 3.**
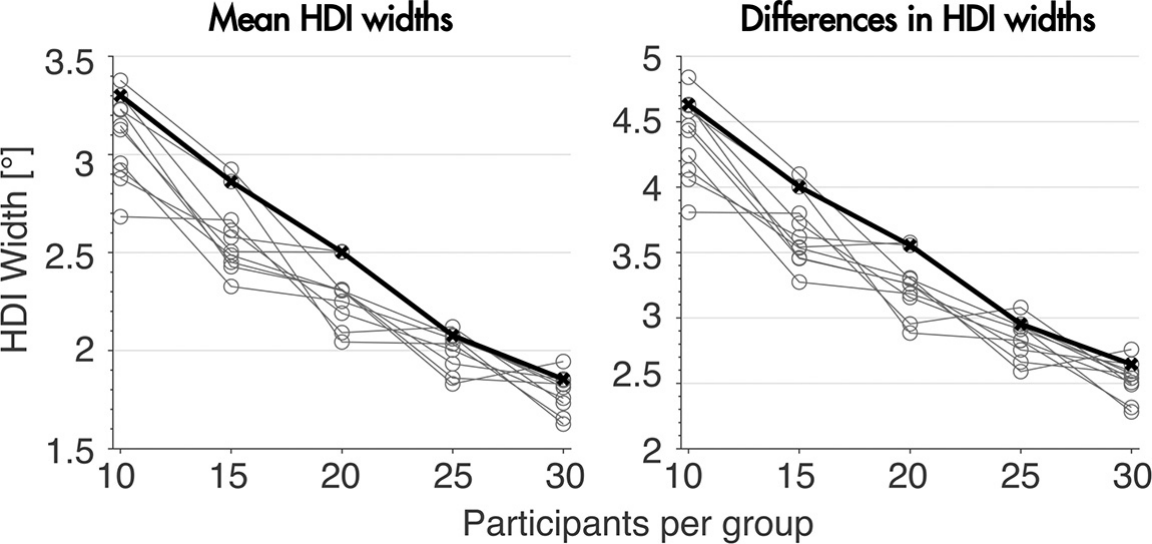
HDI widths as a function of sample size. Number of subjects refers to the number of subjects per group. The black line denotes the 90th percentile of the 10 model repetitions. The left figure shows the HDI width for each group mean, the right figure shows the HDI width for the group differences.

To estimate the sample size necessary, we used the same ANOVA model described above. Using the samples of the model of the first round of data collection (12, 12, and 13 participants for the short duration, medium duration, and long duration groups, respectively), we created 10 new, random datasets for five different sample sizes: 10, 15, 20, 25, and 30 participants per group. Datasets were created using the posterior predictive distribution based on the analysis of our original data. This rendered 5 × 10 datasets with increasing samples sizes. For each of these datasets, we sampled new posterior distributions using the original ANOVA model. The posterior distributions gave us estimates of credible values given our initial model. Next, we calculated the HDIs and HDI widths for the early aftereffect episode from the posterior distributions for each group and for the differences in aftereffect for short duration and medium duration groups ( $\Delta_{\text{SD},\text{MD}}$), for short duration and long duration groups ( $\Delta_{\text{SD},\text{LD}}$), and for medium duration and long duration groups ( $\Delta_{\text{MD},\text{LD}}$) within each dataset and compared them for the different sample sizes. We used this initial dataset to estimate the sample size needed to reach a precision criterion of 2° for the mean per group and 2.5° for the differences between groups. HDI widths were calculated using an ANOVA model based on the initial dataset. As shown in Figure 3, HDI width steadily decreased with increasing sample sizes. We reached our predefined precision criterion of 2° for the mean at 25 subjects per group. Adding five more subjects (30 subjects per group) further reduced the HDI width of the mean only marginally. Although our model predicted that we needed 30 subjects per group to achieve an HDI width of 2.5° for the group differences, our actual data showed that 25 subjects per group were sufficient to reach this precision criterion (see next paragraph). We thus collected our full data set with 78 subjects (27, 25, and 25 for the short duration, medium duration, and long duration groups, respectively).

Full details of the sampling, the posterior samples, creation of the random datasets and code to produce all the diagnostic plots are available in the online repository (https://osf.io/f6smc/).

## Results

The goal of our study was to investigate whether different feedback durations would lead to changes in the amount of implicit contributions to motor adaptation, which was measured by means of the aftereffect. We mainly focused on early aftereffect which we defined as the first 20 aftereffect trials following adaptation. However, we also present the results of the late aftereffect phase, which includes the last 20 aftereffect trials. Our initial dataset consisted of 35 subjects (12, 12, and 13 participants for the short duration, medium duration, and long duration groups, respectively). Results for this initial dataset can be found in the online repository. With this limited sample size, participants in the short duration group seemed to learn faster and reach a higher asymptote as compared with participants in the medium duration and long duration groups. Both during early and late baseline participants in all groups showed a slight offset in counterclockwise direction (−1.5 ± 1.8°, −1.3 ± 1.8°, and −1.7 ± 2° for short duration, medium duration, and long duration during early baseline and −1.4 ± 1.9°, −0.8 ± 1.8°, and −2.1 ± 2° for short duration, medium duration, and long duration during late baseline). We found no differences between groups for our main outcome measure early aftereffect (9.1 ± 2.7°, 6.8 ± 2.6°, and 8.6 ± 2.9° for short duration, medium duration, and long duration, respectively) and for late aftereffect (8.6 ± 2.6°, 5.4 ± 2.5°, and 8.5 ± 2.8° for short duration, medium duration, and long duration, respectively). However, HDI widths were all above 2.5° in this initial dataset, which is 0.5° higher than our precision criterion (see Materials and methods, Sample size estimation) meaning that a larger sample was needed to achieve a reasonable HDI width.

### Final dataset

As predicted by our sample size estimation, HDI widths of mean movement directions in our final dataset were below 2°. For the three groups HDI widths were 1.8°, 1.7°, and 1.8° during early aftereffect and 1.8°, 1.7°, and 1.9° during late aftereffect. HDI widths of the differences between groups (presented as $\Delta_{\text{SD},\text{LD}}$, $\Delta_{\text{SD},\text{MD}}$, and $\Delta_{\text{MD},\text{LD}}$) were 2.4, 2.5, and 2.6 during early aftereffect and 2.5, 2.6, and 2.5 during late aftereffect.

**Figure 4.**
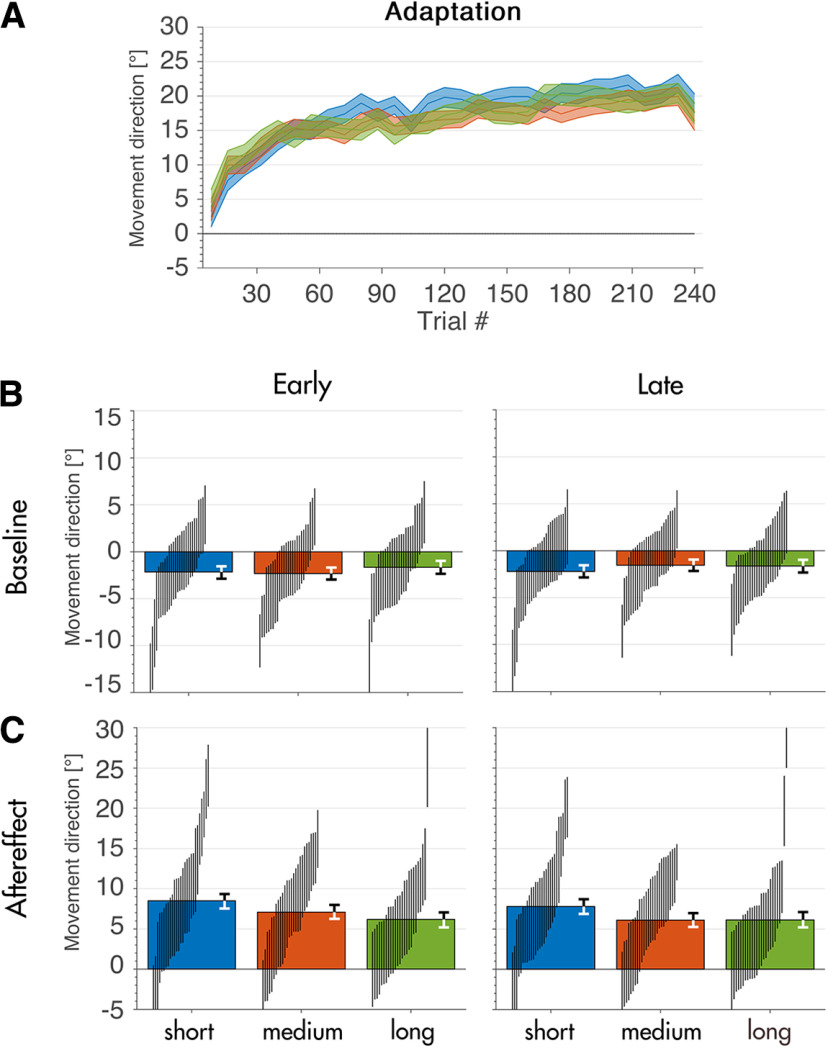
Learning curve and aftereffect. ***A***, Binned learning curve for all three groups (30 bins of eight trials each). Shaded area denotes 95% HDI. ***B***, Mean movement direction during early and late baseline for all three groups. ***C***, Mean movement direction during early and late aftereffect for all three groups. Error bars show 95% HDI for each group, vertical lines show 95% HDI for individual subjects.

**Figure 5.**
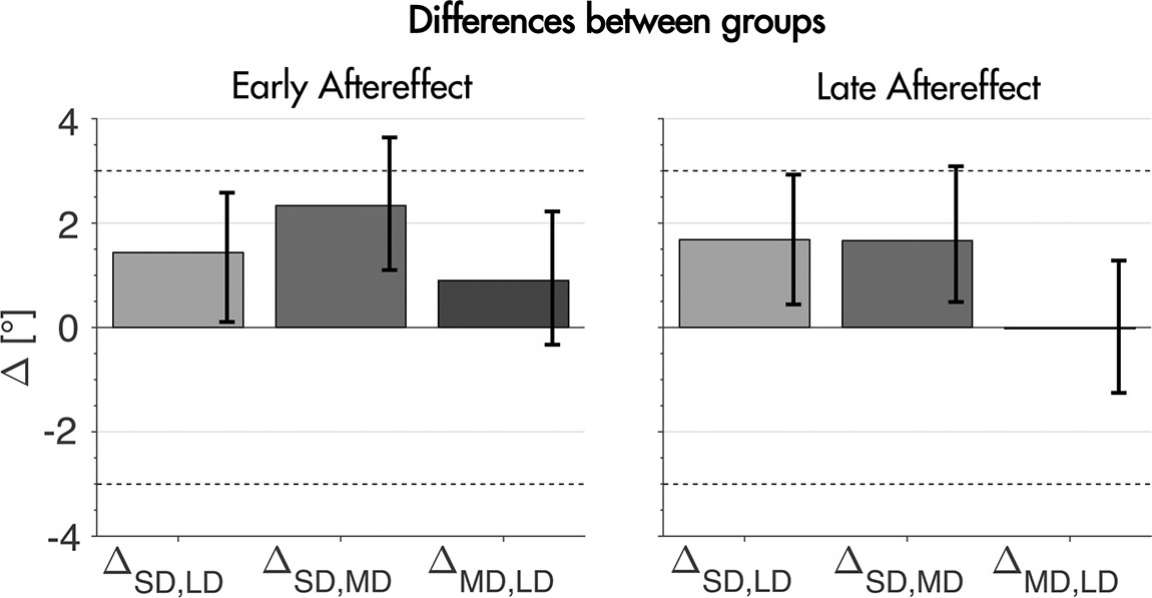
Differences between groups during early and late aftereffect. ΔSD,LD denotes the difference between the short duration and the long duration group, ΔSD,MD shows the difference between short duration and medium duration and ΔMD,LD the difference between the medium and the long duration group. Δ[°] is the difference in degrees. Dashed horizontal lines represent the ROPE of −3% through 3% around a value of 0 difference between the groups. Error bars show 95% HDI for the group differences.

The time course of movement directions in our final dataset showed a stereotypical learning curve for all three groups (Fig. 4*A*). Contrary to our observations in the initial data set, asymptote measured as the mean of the last three bins (24 trials) was comparable across groups (20.3 ± 1.4°, 18.7 ± 1.3°, and 19.3 ± 1.5° for short duration, medium duration, and long duration). Movement direction during early and late baseline was similar to the outcomes in our initial dataset where participants in all groups showed a small offset in counterclockwise direction (−2.2 ± 1.3°, −2.3 ± 1.3°, and −1.7 ± 1.4° for short duration, medium duration, and long duration during early baseline and −2.2 ± 1.3°, −1.6 ± 1.2°, and −1.6 ± 1.3° for short duration, medium duration, and long duration during late baseline; Fig. 4*B*). Movement direction during early aftereffect and late aftereffect also stayed similar to our results in the initial dataset, however with a higher precision (8.5 ± 1.8°, 7.1 ± 1.7°, and 6.2 ± 1.8° for short duration, medium duration, and long duration during early and 7.8 ± 1.8°, 6.1 ± 1.7°, and 6.1 ± 1.9° for short duration, medium duration, and long duration during late aftereffect). Baseline correction (because of the offset we found) of adaptation and aftereffect trials did not change these results (data not shown). Differences between the groups were smaller than 2° for all differences except the difference between the SD and the LD group during early aftereffect (Fig. 5). We present the differences in aftereffect for short duration and medium duration groups ( $\Delta_{\text{SD},\text{MD}}$), for short duration and long duration groups ( $\Delta_{\text{SD},\text{LD}}$), and for medium duration and long duration groups ( $\Delta_{\text{MD},\text{LD}}$) as described above and shown in Figure 5. For early aftereffect, the differences were small and included 0 in all cases ( $\Delta_{\text{SD},\text{MD}}$: 1.4 ± 2.4°, $\Delta_{\text{SD},\text{LD}}$: 2.3 ± 2.5°, and $\Delta_{\text{MD},\text{LD}}$: 0.9 ± 2.6°). The HDI and the ROPE overlapped for the first and second difference; however, this overlap reflected only 0.01% of the posterior density for $\Delta_{\text{SD},\text{MD}}$. For $\Delta_{\text{SD},\text{LD}}$, this overlap reflected 15% of the posterior density. Considering the small differences we found, we believe this overlap to be negligible. For late aftereffect, the differences between groups were $\Delta_{\text{SD},\text{MD}}$: 1.7 ± 2.5°, $\Delta_{\text{SD},\text{LD}}$: 1.7 ± 2.6°, and $\Delta_{\text{MD},\text{LD}}$: −0.1 ± 2.6°, the HDIs included 0 in all cases. As during early aftereffect, the HDI and ROPE overlapped for $\Delta_{\text{SD},\text{MD}}$ and $\Delta_{\text{SD},\text{LD}}$; however, this overlap corresponded to only 0.02% of the posterior density for both differences.

Finally, we found no differences between groups in movement times (430 ± 130, 420 ± 160, and 430 ± 130 for short duration, medium duration, and long duration), peak velocities (6.5 ± 2.4, 6.5 ± 3.3, and 6.6 ± 2.6 m/s for short duration, medium duration, and long duration) and reaction times (680 ± 370, 810 ± 280, and 790 ± 320 ms for short duration, medium duration, and long duration).

## Discussion

The purpose of this study was to investigate whether changes in feedback duration in a visuomotor rotation task lead to changes in implicit recalibration. Specifically, we hypothesized that shorter feedback durations may lead to more implicit recalibration, possibly by weakening the association between hand and visual cursor by means of enhanced reliance on felt sense of hand position instead of the actual seen hand position. We tested this hypothesis by dividing participants into three groups and presenting them with different feedback durations (200, 600, and 1200 ms). Our measure of implicit recalibration was early aftereffect, which we defined as the first 20 trials after the last rotation trial. We determined that a sample size of 25 participants per group (75 participants in total) would allow us to estimate early aftereffect with an HDI width per group of 2°, which we deemed to be sufficiently small to demonstrate whether or not feedback duration affects implicit recalibration. Using this sample size, we found differences <2° with HDIs including 0 in all cases during early and late aftereffect. During early aftereffect, the difference between the SD and LD group’s ROPE included 15% of the posterior density, meaning that 85% of the posterior distribution of the difference was practically equivalent to zero while 15% of the posterior distribution of the difference fell outside of zero. By traditional standards, 85% of the posterior distribution within the ROPE is not sufficient to demonstrate equivalence. Thus, by these standards, are results remain merely suggestive. However, we strongly suspect that there is no meaningful effect of duration on implicit recalibration for three reasons: (1) the mean difference between SD and LD is very small (only 2.3°); (2) our arbitrarily chosen ROPE was quite conservative and other reasonable choices (e.g., from −3.5° to 3.5°) would have passed the arbitrary traditional criterion for equivalence; and (3) the groups are equivalent in the late aftereffect even under the current ROPE using traditional thresholds. Thus, while the result does require replication, our results do suggest that changes in feedback duration may not affect implicit recalibration.

Our study is the first to directly compare different feedback durations and their effect on implicit recalibration in a visuomotor rotation task. One other study included two different feedback durations (350 and 700 ms) as secondary comparison in one of their experiments, however, they did not find any differences between these two conditions and only presented the averaged outcomes (Bromberg et al., 2019). In their second experiment, Bromberg et al. (2019) present visual feedback for 1000 ms. This is also the case for the majority of studies using endpoint feedback (Taylor et al., 2014; Schween and Hegele, 2017; de Brouwer et al., 2018). Other studies chose longer durations: Bond and Taylor (2015) presented participants with feedback for 1500 ms and Schween et al. (2014) showed feedback for 1600 ms. However, since these studies set out to compare different manipulations of the task (such as rotation size, reporting vs nonreporting or online vs endpoint feedback), which all have large effects on adaptation, it is difficult to draw conclusions about possible effects of the different durations from them.

There is previous evidence that manipulations of visual feedback affect different parameters in adaptation tasks. In prism adaptation, for instance, delaying visual feedback from early to late in movement has been shown to increase adaptation, and thus performance, in general (Redding and Wallace, 1990) and explicit re-aiming in particular (Redding and Wallace, 1992, 1994; Redding et al., 2005). Feedback duration, on the other hand, did not seem to affect any parameters of adaptation (Redding and Wallace, 1990). In visuomotor rotations, timing of visual feedback throughout the task has also been shown to affect performance: when visual feedback is provided at the beginning of a reach, even if this early presentation was as brief as 45 ms, endpoint precision is improved in a simple reaching task. However, if visual feedback is presented later, after peak limb velocity, online corrections are less efficient with high temporal costs (Kennedy et al., 2015; Tremblay et al., 2017). This stands in line with the findings in prism adaptation and indicates that even minimal feedback durations, if presented early during the movement, lead to optimal performance. In contrast, delaying endpoint feedback while keeping feedback durations constant at 1000 ms, which is similar to delaying visual feedback to late in movement in prism adaptation, has been shown to decrease implicit recalibration in visuomotor rotations (Kitazawa et al., 1995; Brudner et al., 2016; Schween and Hegele, 2017). From this it seems that delayed visual feedback and duration of visual feedback should both affect performance and implicit recalibration. So, why did not we find an effect of prolonged feedback durations on adaptation?

We see two possibilities. One is that extending visual feedback at the end of the movement does not strengthen the association between the hand and the visual cursor (contra our hypothesis). The second is that the association is strengthened, but that it does not affect implicit recalibration. We find the second possibility unlikely: a large number of studies has shown proprioceptive recalibration to take place in visuomotor rotation tasks (van Beers et al., 2002; Simani et al., 2007; Cressman and Henriques, 2009). Proprioceptive recalibration results from the discrepancy between our estimates of hand position based on proprioceptive inputs and those based on visual input (Cressman and Henriques, 2009; Cressman et al., 2010; Ruttle et al., 2016) and is generally viewed as an implicit process (Hinder et al., 2008; Modchalingam et al., 2019; Tsay et al., 2021). However, since measuring proprioceptive recalibration was not part of our setup, we cannot validate whether changes occurred or not.

Thus, we suspect that long duration visual feedback at the end of movement does not strengthen the association of hand to cursor. Perhaps it would if it were presented earlier or if we waited to present visual feedback until the hand came to a stop. We presented feedback when participants reached a distance of 7.6 cm from the target, so their hand was still moving while feedback was stationary. This is a common way of presenting endpoint feedback (Taylor et al., 2014; McDougle et al., 2015; de Brouwer et al., 2018; Bromberg et al., 2019), but it may have undermined the potential for developing an association.

One additional limitation of our study is that we restricted our attention to visual stimuli for which we expect fully conscious sensory processing. We do not know whether our results would generalize to feedback durations shorter than 200 ms. The question of how adaptation to visuomotor rotation is affected by the use of stimuli that are below or near the threshold for conscious processing is an interesting question that has not been addressed in the literature.

These and other limitations of this study should be addressed in future research. We focused on implicit recalibration as measured by the aftereffect, and feedback duration may affect other parameters of adaptation that we did not measure. For instance, we did not test for proprioceptive recalibration, as pioneered by Cressman and Henriques (2009). We also did not test for changes in explicit re-aiming. Although it is commonly assumed that explicit re-aiming and implicit recalibration sum up to the total adaptation, this may not be the case (Kim et al., 2019; McDougle and Taylor, 2019; Maresch et al., 2020, 2021). If it is not, explicit re-aiming could be affected by feedback duration without an observable change in implicit recalibration.

In sum, this research provides new evidence about the importance of feedback duration in visuomotor rotations. Our results, based on a large sample size, suggest that implicit recalibration may not be affected by longer visual feedback. This work should be extended by examining how visual feedback duration affects other parameters of adaptation and whether other forms of degraded visual feedback may influence implicit recalibration or any of the other parameters. Last, we would also, once again, like to point out the importance of a large enough sample size. Early results were strongly suggestive (results of initial dataset can be found in the online repository). However, our final dataset convinced us that the effect is not there.

## Synthesis

Reviewing Editor: Ifat Levy, Yale University School of Medicine Decisions are customarily a result of the Reviewing Editor and the peer reviewers coming together and discussing their recommendations until a consensus is reached. When revisions are invited, a fact-based synthesis statement explaining their decision and outlining what is needed to prepare a revision will be listed below. The following reviewer(s) agreed to reveal their identity: NONE.

Thank you for clarifying the points from the previous round of review. The rewording of the research hypothesis is now clearer and less ambiguous.

There are two remaining issues that are worth addressing at this stage:

1. From the description of the multi-level model, the reader may deduce that subject was treated as a fixed effect - please clarify whether it was included as a random effect (as it should, and as the code suggests).

2. Following up on the elaborations in the discussion, it may be worth mentioning the possibility that feedback delays and temporal perturbations of the visual feedback in general may be selectively targeting explicit learning, rather than directly impacting implicit learning, because the processing of information regarding the duration of the visual error could be simply outside of the implicit domain / circuitry (for example see morehead 2017, morehead & de Xivry 2021).

Minor edits:

1. Please use same color for error bars in figure 4 (all black)

2. There is typo in the caption of figure 2: “the left group shows the HDI width for the group differences.” should probably read “the right figure/panel shows the HDI width for the group differences.”

## Author Response

We would again like to thank the reviewers for their time and effort in reviewing our revised manuscript. We respond to the remaining comments in the order of their appearance. The page numbers refer to the pages of the revised manuscript with markup (changes bold face and highlighted in red) with page 1 starting at the introduction.

1. From the description of the multi-level model, the reader may deduce that subject was treated as a fixed effect - please clarify whether it was included as a random effect (as it should, and as the code suggests).

Bayesian statistics does not define fixed and random effects in the same way that frequentist hypothesis testing does and, to speak generally, the distinction is not made. Rather, the structure of the variance is modelled directly in the generative model used to estimate all of the parameters. In our case, the between subject variance was modeled hierarchically. An effort to understand fixed and random effects from a Bayesian perspective is available in this paper:

https://onlinelibrary.wiley.com/doi/full/10.1111/j.1468-0084.2012.00700.x?casa_token=C01P0NNHqmoAAAAA%3AESk28RMgOES2qhRxxV7Sk600whznmcA-UTKfpC74jkV1AhKOd9Nx6O30Cz8_qSvvIU8zwOINCAs

They conclude: “It is usually believed that fixed and random effects estimators are the result of different estimation methods. This paper has shown that, rather than the method or approach adopted, it is the difference in information content captured by the prior distribution of individual effects used in the estimations that produces different slope estimators. In other words, by choosing an estimation method, a Classical econometrician is actually choosing a prior distribution of effects, like her Bayesian colleague. The important point here is that the slope estimator is invariant to the introduction of information on effects”.

We agree that this is an important difference between frequentist statistics and Bayesian statistics and a potential source for confusion. We therefore added a short description clarifying this crucial difference to our manuscript, and we now also reference the above mentioned paper.

2. Following up on the elaborations in the discussion, it may be worth mentioning the possibility that feedback delays and temporal perturbations of the visual feedback in general may be selectively targeting explicit learning, rather than directly impacting implicit learning, because the processing of information regarding the duration of the visual error could be simply outside of the implicit domain / circuitry (for example see morehead 2017, morehead & de Xivry 2021).

We would be happy to include these additional references, however, we are not sure we understand how the reviewer relates them to what is written here. Both papers discuss how different types of errors affect adaptation and its underlying processes. Morehead and de Xivry 2021 moreover beautifully summarize the complexity of the different underlying processes and errors and they succeed in unifying different threads of research in visuomotor rotations. However, we couldn’t find references to paradigms using feedback delays or other kinds of temporal perturbations of visual feedback in their manuscript. Even more so, we found evidence for the opposite statement to be true: feedback delays - whether during the movement or at movement end - have so far only been found to selectively target implicit re-aiming and, except for the study of Schween 2017, they have been shown to have no effect on explicit re-aiming (see Brudner 2016 for feedback delays, Schween 2014 and Bond 2015 for online vs endpoint feedback in visuomotor rotations and Maddox 2003 for feedback delays in category learning). We have thus decided not to include the references suggested by the reviewer.

Minor edits:

1. Please use same color for error bars in figure 4 (all black)

We have changed the color of the error bars to black.

2. There is typo in the caption of figure 2: “the left group shows the HDI width for the group differences.” should probably read “the right figure/panel shows the HDI width for the group differences.”

Thank you for pointing out this typo. We have corrected it now
